# Characterization of mRNA Profiles of Exosomes from Diverse Forms of M2 Macrophages

**DOI:** 10.1155/2020/1585306

**Published:** 2020-11-21

**Authors:** Yuan Yue, Suiqing Huang, Zixuan Wu, Keke Wang, Huayang Li, Jian Hou, Xiaolin Huang, Li Luo, Quan Liu, Zhongkai Wu

**Affiliations:** ^1^Department of Cardiac Surgery, The First Affiliated Hospital of Sun Yat-sen University, China; ^2^NHC Key Laboratory of Assisted Circulation, Sun Yat-sen University, China; ^3^Organ Transplant Center, The First Affiliated Hospital of Sun Yat-sen University, China

## Abstract

Exosomes transmit certain amounts of molecules to specific recipient cells for intercellular communication. Among these molecules, messenger RNAs (mRNAs) may be delivered and translated into proteins in the recipient cells, and these mRNAs are thought to be critical mediators of exosomal functions. There are three subtypes of M2 macrophages (M_2_Ф), M_2a_Ф, M_2b_Ф, and M_2c_Ф, which have different specific functional programs. The aim of the present study was to screen the mRNA profiles in the exosomes of these macrophage subtypes and to analyze the transcriptomic profile features associated with their specific functions. The mRNA contents of the exosomes isolated from the culture supernatants of the M_2_Ф subtypes were analyzed and compared using the Illumina HiSeq platform. The results indicated that the exosomes contained particular mRNAs from their source cells and were messengers of cellular functions. Bioinformatics analysis suggested that the exosomal mRNAs from M_2b_Фs are enriched in the Toll-like receptor (TLR), tumor necrosis factor (TNF), NOD-like receptor (NLR), and NF-kappa B (NF-*κ*B) signaling pathways. The mRNA profile of exosomes from M_2b_Ф was distinctly different from that of exosomes from M_2a_Ф and M_2c_Ф and was consistent with the M_2b_Ф cytological characteristic of maintaining a high level of proinflammatory cytokine and regulatory factor production. Therefore, the mRNA profiles revealed several characteristics of the exosomes from diverse forms of M_2_Ф. Further functional investigations based on these results may advance the understanding of the physiological roles of exosome-transferred mRNAs in MФ functions.

## 1. Introduction

Exosomes are small membrane-derived vesicles of endocytic origin that are secreted by most cells and are approximately 40–200 nm in diameter [1]. Exosomes are extracellular phospholipid nanocarriers that function as signalosomes and transmit molecules (DNA, RNA, proteins, and lipids) to specific recipient cells for intercellular communication [2, 3]. Among the molecules contained in exosomes, messenger RNAs (mRNAs) may be delivered and translated into proteins in recipient cells [3, 4]. Valadi et al. [3] first reported that exosomes mediate the transfer of mRNAs and demonstrated that exosomes are capable of shuttling mRNAs between mast cells. In addition, Zomer et al. [5] reported that exosomes are able to deliver the mRNA encoding Cre recombinase to recipient cells to perform Cre-LoxP-mediated recombination. Therefore, mRNAs are thought to be critical mediators of exosomal functions.

Macrophages (MФs) are frontier soldiers of the innate immune system, which is mainly composed of epithelial cells, phagocytes, and natural killer (NK) cells [6, 7]. MФs are also widely distributed immune cells that have an indispensable role in pathogen elimination, tissue development, and wound repair [8]. MФs have a high degree of plasticity and may be polarized by the microenvironment to mount specific functional programs. Polarized MФs may be classified into two subtypes: classically activated macrophages (M_1_Фs) and alternatively activated macrophages (M_2_Фs) [9]. M_1_Фs, also called proinflammatory macrophages, are induced by Th1 cytokines or by lipopolysaccharide (LPS) recognition and release high levels of proinflammatory cytokines [10, 11]. M_1_Фs participate in the removal of pathogens, debris clearance, and neovascularization [12]. M_2_Фs, named anti-inflammatory macrophages, are polarized by Th2 cytokines and characterized by high levels of anti-inflammatory cytokines and profibrogenic factors [12, 13]. M_2_Фs may be further subdivided into M_2a_Фs (stimulated by interleukin- (IL-) 4 and IL-13), M_2b_Фs (stimulated by immune complexes (IC) in combination with lipopolysaccharide (LPS)), and M_2c_Фs (stimulated by IL-10, 13, and 14). Although these three phenotypes are all classified as M_2_Фs, they have distinctly different characteristics. M_2a_Фs, also named wound-healing macrophages, mainly secrete profibrotic factors, such as transforming growth factor-*β* (TGF-*β*), to contribute to tissue repair [15]. M_2c_Фs exhibit robust anti-inflammatory activities by releasing large amounts of IL-10 and profibrotic activity by secreting high levels of TGF-*β* [16]. M_2b_Фs, also known as regulatory macrophages, express both proinflammatory and anti-inflammatory cytokines (IL-1*β*, IL-6, tumor necrosis factor- (TNF-) *α*, and IL-10) and regulate the immune response and inflammatory reaction [14, 17, 18]. Furthermore, it was previously reported by our group that M_2b_Фs are able to reduce the activation of cardiac fibroblasts, which is different from the profibrotic actions of M_2a_Фs and M_2c_Фs [19].

Exosomes are able to deliver mRNAs encoding certain crucial proteins to recipient cells, and exosomes from forms of M_2_Фs may partly explain their specific functions. In the present study, the characteristics of the transcriptomic profiles of mRNAs contained in exosomes from polarized M_2_Фs were evaluated. Microarray and bioinformatics approaches were utilized to identify specific genes and to analyze the mRNA expression features. The present study provided one solution for studying how macrophages influence the downstream cells and provide a foundation for further exploring the function of macrophage phenotypes.

## 2. Materials and Methods

### 2.1. Animals

A total of 20 Sprague-Dawley (SD) rats (6-8 weeks, weight 200-250 g) were obtained from Beijing Vital River Laboratory Animal Technology Co., Ltd. All of the animals were allowed free access to food and water and were housed under a constant temperature (22 ± 2°C) and humidity (45 ± 5%) and a 12 h light/dark cycle. All of the animal procedures were approved by the Institutional Animal Care Committee of Sun Yat-sen University and conformed to the Guide for the Care and Use of Laboratory Animals by the US National Institute of Health (NIH).

### 2.2. Isolation and Polarization of MФs

The rats that were used to harvest the MФs were sacrificed by dislocation of the cervical vertebrae. Bone marrow was collected from the tibias and femurs, washed with complete Roswell Park Memorial Institute- (RPMI-) 1640 medium (Gibco, Thermo Fisher Scientific, Waltham, MA, USA), and centrifuged at 500 x g for 5 min. The cells were then cultured in flasks (Corning, NY, USA) at 37°C in a 5% CO_2_ incubator in RPMI-1640 for the first 3 days and then in Dulbecco's modified Eagle's medium (DMEM; Gibco) for the next 3 days to generate mature bone marrow-derived macrophages (BMDMs). Both the RPMI-1640 and DMEM were supplemented with 10% fetal bovine serum (FBS; Gibco) and 10 ng/ml macrophage colony-stimulating factor (MCSF; PeproTech, Rocky Hill, NJ, USA). On day 6, the BMDMs were replated in 24-well plates (Corning) to differentiate into M_1_Фs with the addition of 1 *μ*g/ml LPS (Sigma-Aldrich, St. Louis, MO, USA), into M_2a_Фs with 20 ng/ml IL-4 (PeproTech) and 20 ng/ml IL-13 (PeproTech), into M_2b_Фs with 50 *μ*g/ml IgG (Sigma-Aldrich) and 100 ng/ml LPS (Sigma-Aldrich), or into M_2c_Фs with 20 ng/ml IL-10 (PeproTech) [20–23]. After incubation for 24 h, the cells were harvested for marker analysis or the cells were replated and cultured with fresh FBS-free DMEM without stimulation for another 24 h. Subsequently, the cell-free supernatants were collected at 24 h for harvesting of exosomes.

### 2.3. Identification of MФs by Flow Cytometry and Reverse Transcription-Quantitative PCR (RT-qPCR)

The BMDMs were harvested and prepared as single-cell suspensions after 5 min of digestion by 0.25% trypsin (Gibco) at 37°C. Subsequently, the purified cells were stained with APC-Cy7-conjugated anti-rat CD45 antibody (cat. no. 561588; BD Biosciences, San Jose, CA, USA) and FITC-conjugated anti-rat CD68 antibody (cat. no. MA5-28262; Invitrogen, Carlsbad, CA, USA) or stained with isotype control (cat. no. 557873, BD Biosciences and cat. no. 11-4714-81, Invitrogen). Flow cytometry was performed using a Beckman CytoFLEX Flow Cytometer (Beckman Coulter, Miami, FL, USA), and the results were analyzed with FlowJo software (FlowJo, Ashland, OR, USA). The total RNA of the cells was extracted using TRIzol reagent (Invitrogen), and complementary (c) DNA was synthesized using the qPCR RT Master Mix kit (TOYOBO, Osaka, Japan). PCR primers were designed and synthesized by Invitrogen (Thermo Fisher Scientific). qPCR analysis was performed according to the manufacturer's protocols using the KOD SYBR qPCR Mix (TOYOBO) in a LightCycler 480 (Roche, Basel, Switzerland). For analysis, the expression of target genes was normalized to that of GAPDH. The primer sequences for the target genes are illustrated in Table 1.

### 2.4. Isolation of Exosomes from the MФ Culture Supernatants

The culture supernatants containing exosomes were centrifuged at 2,000 x g and 4°C for 10 min to remove the cells and debris. Subsequently, the exosomes were extracted and collected from the culture supernatants with an exoEasy Maxi Kit (Qiagen GmbH, Hilden, Germany) according to the manufacturer's protocol. In short, the exoEasy Maxi Kit uses a membrane-based affinity binding step to isolate exosomes from cell culture supernatants. The method does not distinguish exosomes by size or cellular origin and is not dependent on the presence of a particular epitope. Instead, it makes use of a generic, biochemical feature of exosomes to recover the entire spectrum of exosomes present in samples. Particulate matter other than exosomes, like larger protein complexes, is largely removed during the binding and ensuing wash step. After washing the column membrane, intact exosomes are eluted in an aqueous buffer containing primarily inorganic salts and are then ready to use for the subsequent analysis.

### 2.5. Transmission Electron Microscopy Observations and qNano Analysis of Exosomes

The exosome samples were added to the copper mesh and precipitated for 3 min. Phosphotungstic acid was used for negative staining. Finally, the mesh was observed with a transmission electron microscope (JEM-1200EX; JEOL, Tokyo, Japan). The sizes and concentrations of the exosomes were determined using qNano analysis (Izon Science, Burnside, Christchurch, New Zealand) based on the manufacturer's instructions.

### 2.6. Western Blot Analysis

The total protein from the cells and exosomes was lysed using RIPA lysis buffer (Beyotime Biotechnology, Shanghai, China). The protein concentrations were measured with a Bio-Rad Protein Assay Kit (Bio-Rad Laboratories, CA, USA). The proteins were fractionated by 8-15% sodium dodecyl sulfate-polyacrylamide gels (SDS-PAGE) and transferred to polyvinylidene fluoride (PVDF) membranes (Millipore, Billerica, MA, USA). The membranes were blocked and then incubated with antibodies against CD9 (cat. no. ab92726; Abcam, Cambridge, MA, USA), heat shock protein (HSP) 70 (cat. no. 66183-1; Proteintech Group, Wuhan, China), tumor susceptibility gene 101 (TSG101; cat. no. ab133586; Abcam), and cytochrome C (cat. no. ab13575; Abcam). Subsequently, the membranes were incubated with a horseradish peroxidase-conjugated secondary antibody (Thermo Fisher Scientific) at room temperature for 1 h and were visualized using enhanced chemiluminescence reagents (Millipore) according to the manufacturer's instructions.

### 2.7. Exosomal RNA Extraction and mRNA Sequencing (mRNA-seq) Library Preparation

The total exosomal RNA was extracted using the exoRNeasy Serum/Plasma Maxi Kit (Qiagen GmbH) according to the manufacturer's protocol and used for mRNA sequencing. RNA quantification was performed with Qubit 3.0 (Thermo Fisher Scientific). For mRNA-seq, library preparation was performed using the Epi™ longRNA Ampli kit (Epibiotek, Guangzhou, China) according to the manufacturer's instructions. In brief, the ribosomal RNA (rRNA) was depleted and the RNA samples were mixed with the provided RT Primer mix and deoxynucleoside triphosphate (dNTP) mix to perform RT and synthesize the first-strand cDNA by PCR. After RT, the second-strand cDNA was synthesized by PCR under optimized reaction conditions. After amplification by PCR, cDNA was purified and size-selected with magnetic beads. Library evaluation and quantification were performed on a Qseq100 Bio-Fragment Analyzer (BiOptic, Taiwan, China), and subsequent next-generation sequencing (NGS) was performed by using an Illumina HiSeq X Ten System (Illumina, San Diego, CA, USA).

### 2.8. Bioinformatics Analysis of the mRNA-seq Data

In brief, adaptor and primer sequences from the library were trimmed. After trimming, the sequence reads were then aligned to the rat genome (version Rnor_6.0) using Hisat2, followed by a postalignment quality check to assess the performance of the alignment. After the alignment, HTseq was used to determine the counts of the reads mapped to the genome. Reads per kilobase per million mapped reads (RPKM) was used to standardize the expression data, which allowed for the comparison of expression levels between mRNAs. Using the DESeq2 algorithm, mRNAs of different abundances were identified by fold change (>2-fold) and significance level (*P* < 0.05) filtering. Volcano plots were drawn using R based on the differential mRNA analysis, and the color was determined based on the filtering criteria. Networks of these mRNAs were algorithmically generated based on the potential connectivity of their products using the Database for Annotation Visualization and Integrated Discovery online database (DAVID; version 6.8; http://david. http://ncifcrf.gov) [24, 25] and Cytoscape (version 3.6.1) [26], which is an open-source bioinformatics software platform for visualizing molecular interaction networks. Enrichment analysis, including Gene Ontology (GO) functional analysis and Kyoto Encyclopedia of Genes and Genomes (KEGG) pathway analysis, was performed using DAVID. Furthermore, the plug-in ClueGO (version 2.5.5) of Cytoscape was used to search for potential associated pathways of specific genes [27].

### 2.9. Statistical Analysis

Statistically significant differences between groups were assessed by one-way analysis of variance (ANOVA) followed by Bonferroni's post hoc test. *P* < 0.05 was considered to indicate statistical significance. Statistical tests were performed using GraphPad Prism Software (Version X; GraphPad, La Jolla, CA, USA).

## 3. Results

### 3.1. Identification of Different Subtypes of M_2_Ф

**Figure 1:**
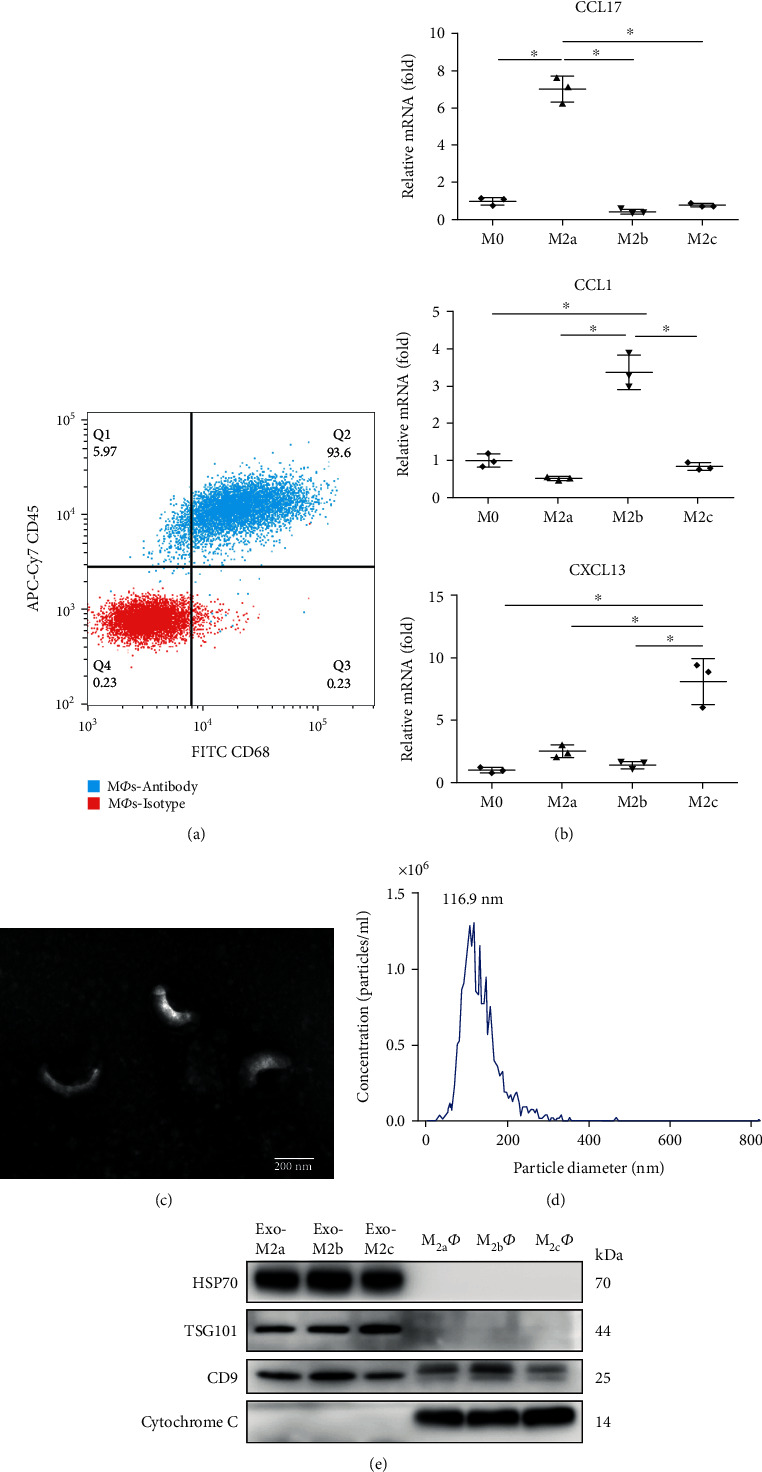
Identification of M_2_Фs and exosomes. (a) Procedure for the identification of BMDMs positive for CD45 and CD68 by flow cytometry. (b) Gene expression profiles of the different M_2_Ф phenotypes measured by reverse transcription-quantitative PCR. M_2a_Фs, M_2b_Фs, and M_2c_Фs expressed high levels of CCL17, CCL1, and CXCL13, respectively. Data are presented as fold changes relative to the expression levels in untreated BMDMs and are the mean of three independent experiments. ^∗^*P* < 0.05. (c) Representative transmission electron microscopy image of exosomes (scale bar, 200 nm). (d) The size and concentration of exosomes measured by qNano analysis. (e) Western blot analysis of exosome marker proteins and negative protein. BMDM: bone marrow-derived macrophage; CCL: C-C motif chemokine ligand; CXCL: C-X-C motif chemokine ligand.

Following the specific treatments of the BMDMs, they were stained and analyzed by flow cytometry to quantify the cells with the expression of CD45 (APC-Cy7) and CD68 (FITC), which are markers for MФs. The results indicated that the bone marrow cells had differentiated into MФs (Figure 1(a)). Subsequently, the phenotypes of the polarized BMDMs were identified based on their gene expression using qPCR. M_2a_Фs, M_2b_Фs, and M_2c_Фs expressed high levels of C-C motif chemokine ligand (CCL) 17, CCL1, and C-X-C motif chemokine ligand (CXCL) 13, respectively (Figure 1(b)). Collectively, these results indicated that BMDMs were successfully polarized into different M_2_Ф subtypes.

### 3.2. Validation of the Exosomes Isolated from the Cell Culture Supernatants

Exosomes were extracted and collected from the cell supernatants with an exoEasy Maxi Kit (Qiagen GmbH). The exosomes were identified using an electron microscope, and they exhibited typical vesicle-like structures and diameters of approximately 100-200 nm (Figure 1(c)). The qNano analysis technique was used to measure the size and concentration of the exosomes (Figure 1(d)). The results indicated that the diameter was 30-400 nm (median, 116.9 nm). By using western blot analysis, well-known exosomal maker proteins, TSG101, HSP70, and CD9, were detected in exosomes, but not in M_2_Ф cells. On the other hand, cytochrome C was detected in M_2_Ф cells, but it was absent from the exosomes, indicating that the exosomes were not contaminated with apoptotic bodies or cell debris (Figure 1(e)). These data indicated that exosomes were acquired from the supernatants with good purity.

### 3.3. An Overview of the mRNA-seq Results

**Figure 2:**
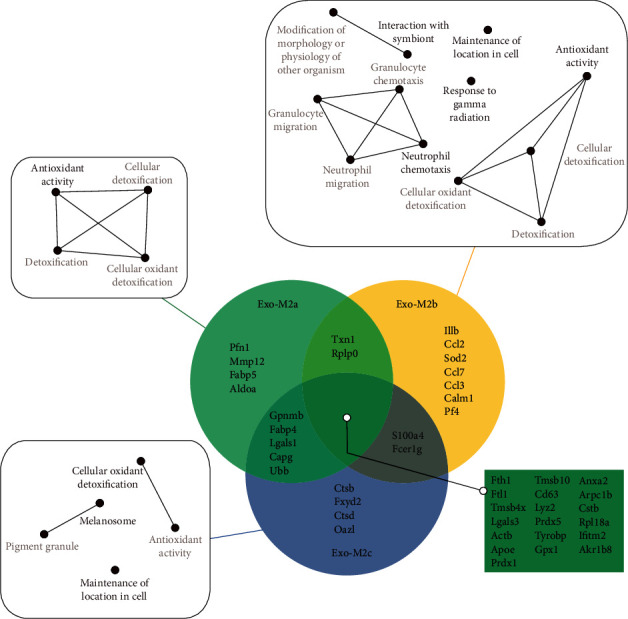
Top 30 most abundant mRNAs in exosomes from different M_2_Фs and the pathway networks. The top 30 mRNAs in the exosomes are presented in the Venn diagram. The GO pathway networks of the mRNAs were categorized using CluePedia. The threshold for the network analysis was set to *P* < 0.05 and FDR < 0.05. GO: Gene Ontology; FDR: false discovery rate.

For clarity, the exosomes from M_2a_Фs, M_2b_Фs, and M_2c_Фs were abbreviated as Exo-M2a, Exo-M2b, and Exo-M2c, respectively. The top 30 mRNAs in the exosomes are presented in a Venn diagram in Figure 2. Fth1, Ftl1, and Tmsb4x were the 3 most abundant transcripts in all of the groups. The other highly abundant mRNAs in all the groups were Lgals3, Actb, Apoe, Prdx1, Tmsb10, and Lyz2. The results indicated that numerous transcripts are abundant in the exosomes from all three phenotypes, while the transcript of Il1b, which encodes IL-1*β*, was preferentially detected in Exo-M2b and was less abundant in Exo-M2a and Exo-M2c. There were other cytokine- and chemokine- associated transcripts that were highly expressed in Exo-M2b but not in Exo-M2a and Exo-M2c; these transcripts included CCL2, CCL7, CCL3, and Pf4. These mRNAs were then categorized into GO pathway networks according to molecular function using CluePedia (Figure 2).

### 3.4. Unique mRNAs Contained in the Exosomes

By analyzing the mRNA profiles of the exosomes, it was revealed that certain mRNAs were detectable in the exosomes from a particular phenotype but barely detectable in the exosomes from the other phenotypes. To identify transcripts unique to these phenotypes, criteria of mRNAs were detected in one group with an RPKM value of >5 and not detected in other groups (RPKM < 0.1). With these criteria, 12, 13, and 1 mRNAs were identified as unique transcripts in Exo-M2a, Exo-M2b, and Exo-M2c, respectively (Table 2).

### 3.5. Profile of Differentially Abundant mRNAs

**Figure 3:**
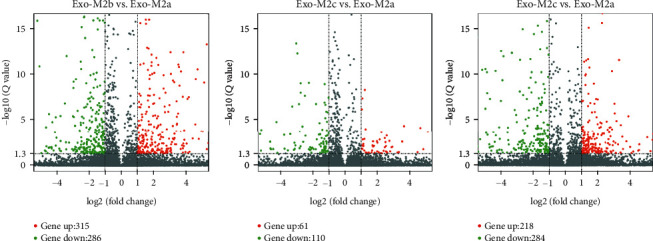
Volcano plot comparing the levels of mRNA abundance between groups. Red and green dots represent upregulated and downregulated mRNAs (>2.0-fold change and *P* < 0.05), respectively.

Based on a threshold set at a fold change of >2 and *P* < 0.05 for the microarray data, a total of 601, 171, and 501 differentially abundant mRNAs were identified in Exo-M2b vs. Exo-M2a, Exo-M2c vs. Exo-M2a, and Exo-M2c vs. Exo-M2b, respectively. Volcano plots were drawn to illustrate the variance in abundance at different *Q* values (corrected *P* value via false discovery rate (FDR) estimation) and fold changes (Figure 3). The top 30 differentially abundant mRNAs in the exosomes between groups are presented in Table 3.

### 3.6. Functional Analyses of mRNAs in the Exosomes

**Figure 4:**
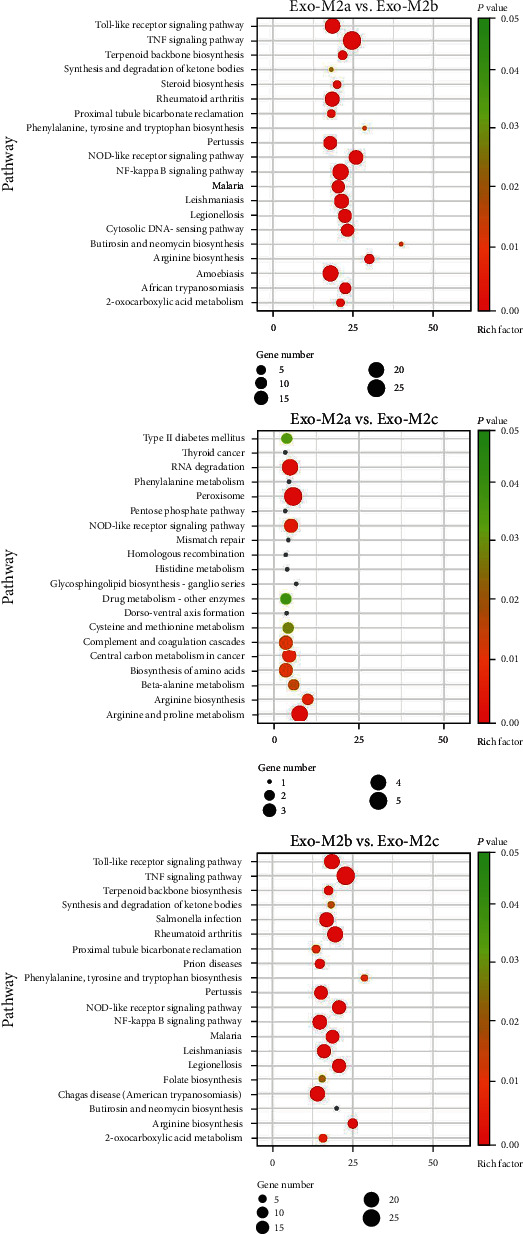
KEGG pathway analysis of the differentially abundant mRNAs between groups. The size of the circles indicates the number of genes involved in the pathway, and the color of the circles represents the *P* value. The threshold for the analysis was set to *P* < 0.05 and FDR < 0.05. KEGG: Kyoto of Encyclopedia of Genes and Genomes; FDR: false discovery rate.

**Figure 5:**
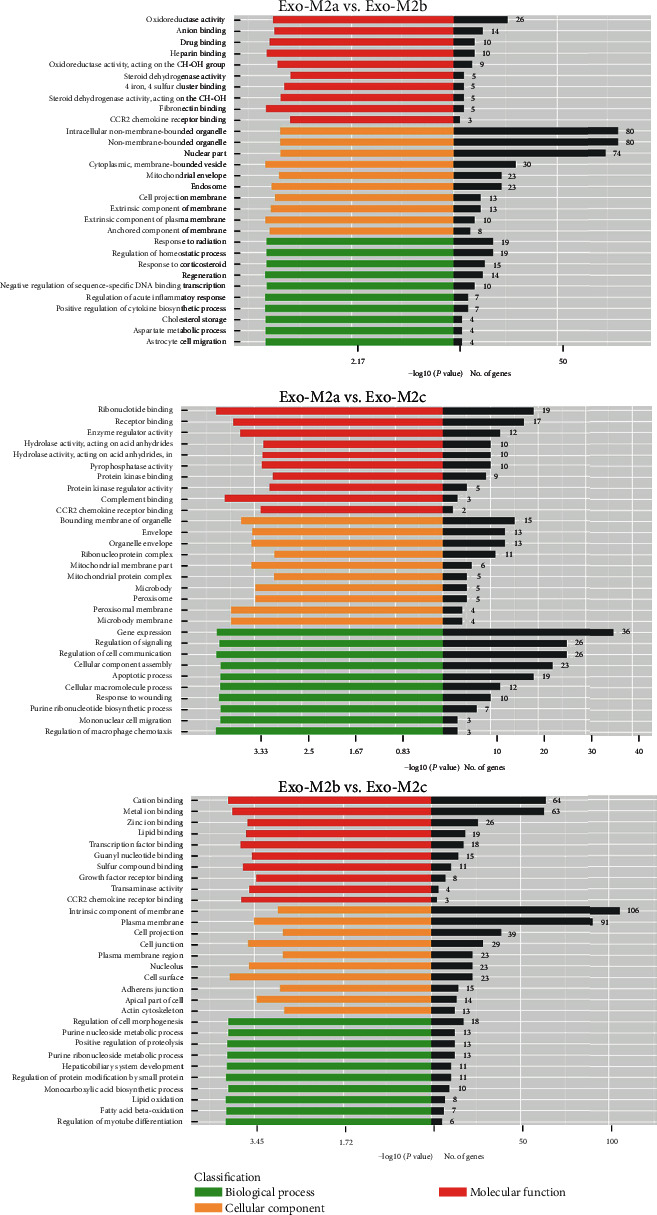
GO analysis of the differentially abundant mRNAs between groups. Bar graphs indicate the number of genes that belong to the category. The threshold for the analysis was set to *P* < 0.05 and FDR < 0.05. GO: Gene Ontology; FDR: false discovery rate.

Given that exosomes contain full-length mRNAs that may be translated to affect biological processes in recipient cells, a Kyoto Encyclopedia of Genes and Genomes (KEGG) pathway enrichment analysis was performed and GO analysis was employed to obtain insight into the potential biological functions of the differentially abundant mRNAs. The mRNA-associated molecules were functionally annotated and characterized, and the higher-level functions were represented by networks of molecular interactions, reactions, and associations, that is, the biological pathways. The major pathways identified by KEGG are presented in Figure 4. KEGG pathway analysis suggested that the different mRNAs between Exo-M2a and Exo-M2b involved in the “Toll-like receptor (TLR) signaling pathway,” “tumor necrosis factor (TNF) signaling pathway,” “NOD-like receptor (NLR) signaling pathway,” “NF-kappa B (NF-*κ*B) signaling pathway,” and “cytosolic DNA-sensing pathway” were enriched. The different mRNAs between Exo-M2b and Exo-M2c were also involved in the first four pathways listed above. Therefore, the exosomes from M_2b_Фs may affect these pathways in recipient cells differently from the exosomes from M_2a_Фs and M_2c_Фs. The different mRNAs between Exo-M2a and Exo-M2c were too disperse to exhibit any involvement in pathways, as indicated by small gene numbers and enrichment factors of pathways. GO analysis identified terms in three categories: biological process, cellular component, and molecular function. According to GO analysis, thousands of GO terms were significantly enriched for different mRNAs among all of the groups, based on thresholds of *P* < 0.05 and FDR < 0.05. The main GO categories are presented in Figure 5.

### 3.7. Profile of mRNAs Involved in the Inflammatory Response

**Figure 6:**
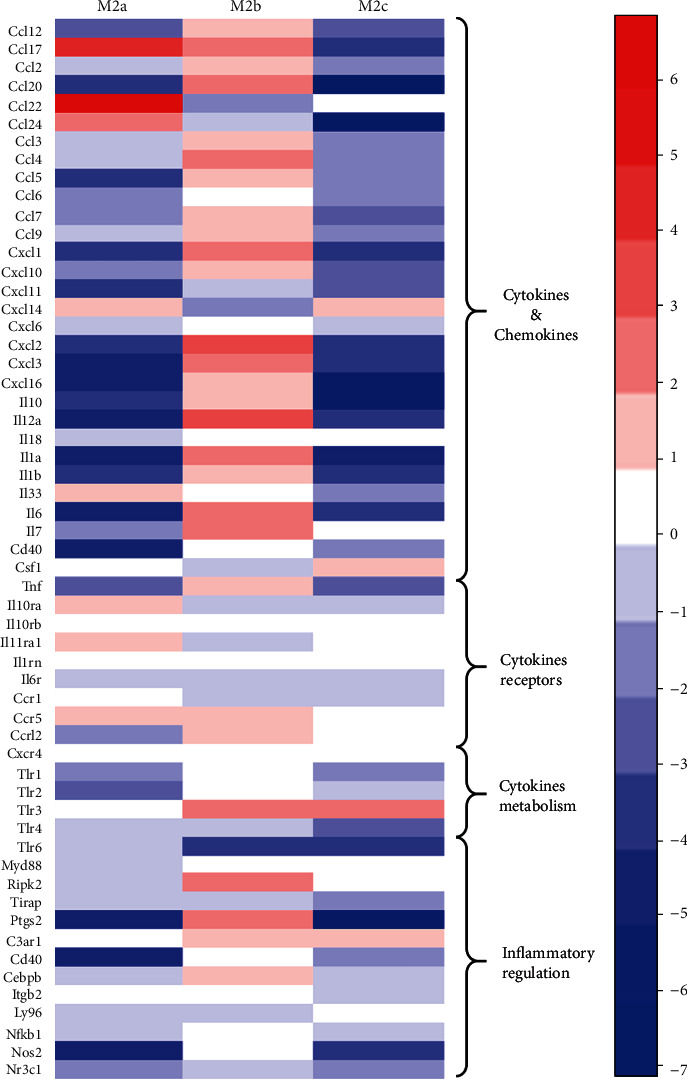
Cluster analysis of the inflammation-associated mRNA profile of the exosomes from the three M_2_Ф subtypes. The ratios presented in this figure were determined by comparison to the mRNA expression of the exosomes from BMDMs. Each column represents the indicated sample; each row indicates a significant fold change in mRNA. Upregulated and downregulated genes are indicated in red and blue, respectively. BMDM: bone marrow-derived macrophages.

The inflammation-associated mRNA profile of the exosomes from the three M_2_Ф subtypes was compared (Figure 6). The ratios presented in the figure were determined by comparing the mRNA profiles of the exosomes from undifferentiated macrophages. The results suggested that numerous transcripts encoding cytokines and chemokines had much higher expression in only the M_2b_Ф population compared with those in the other two populations. Furthermore, the transcript of CCL22 was most abundant in the exosomes from M_2a_Фs. Taken together, these results indicated that exosomes from different populations expressed numerous unique inflammation-associated mRNA transcripts that equip them for specialized local functions.

## 4. Discussion

In the present study, transcriptome analysis of the exosomes from three of M_2_Ф subtypes was performed, focusing on the mRNA profiles. The abundant mRNAs in each subtype and the different mRNAs between subtypes were examined in detail. In general, the present analysis revealed that exosomes contain various mRNAs and have distinct features. The majority of the top 30 abundant mRNAs were identical among the three subtypes. Furthermore, cluster analysis of mRNAs involved in the inflammatory response and bioinformatics analysis demonstrated that the mRNA profile of Exo-M2b was quite different from that of the other two types of exosomes, which is consistent with their different cytological characteristics.

MФs are able to be activated in response to the stimulus they sense, which allows them to combat pathogens, mediate inflammatory reactions, and heal tissue damage in hypersensitivity reactions. In the recent decade, a classification has been developed that describes the complex mechanism of MФ activation as a polarization towards two opposite states, M_1_Ф and M_2_Ф [28]. M_1_Фs, or classically activated MФs, are characterized by inflammatory cytokine secretion and nitric oxide (NO) production, resulting in an effective pathogen killing mechanism [29]. M_2_Фs, or alternatively activated MФs, widely act as anti-inflammatory, proresolving, wound-healing, and trophic or regulatory macrophages [30]. Based on the applied stimuli and the achieved transcriptional changes, M_2_Фs are classified into the M_2a_Ф, M_2b_Ф, and M_2c_Ф subtypes. However, the current classification of the M_2_Ф emphasizes the activation stimuli, rather than the functions [16]. These three subtypes have distinctly different functions in pathophysiological processes.

Prior studies of M_2b_Фs in heart injury and fibrosis by our group reported that they were distinctly different from M_2a_Фs and M_2c_Фs [19, 31]. M_2b_Фs maintained a balance between anti- and proinflammatory functions, which indicated that they were regulatory MФs and were consistent with the findings of Mosser et al. [14, 17]. The impact of the M_2_Ф subtypes, culture supernatants, and exosomes on the activation of cardiac fibroblasts (CFs) was further studied *in vitro.* The results indicated that M_2a_Фs and M_2c_Фs promoted the activation of CFs, while M_2b_Фs showed the opposite effect. Furthermore, the culture supernatants and exosomes showed the same effects as their source cells (Figure S1). These results demonstrated that the exosomes contained important messages from macrophages and transmitted them to downstream cells. Molecules and pathways associated with cardiac fibrosis, including TNF, IL-1, IL-10, TGF family, and platelet-derived growth factors (PDGFs), were might be involved. Therefore, the present study revealed several novel features of the exosomes from M_2_Ф subtypes by focusing on the mRNA profiles.

Several mRNAs encoding cytokines and chemokines, namely, Il1b, CCL2, CCL7, CCL3, and Pf4, were abundant in M_2b_Ф exosomes. The same observation was made in the cluster analysis of genes involved in the inflammatory response, in which M_2b_Ф exosomes contained more transcripts associated with proinflammatory cytokines, chemokines, and regulation factors than M_2a_Ф and M_2c_Ф exosomes. This characteristic was also reported in M_2b_Фs, which were indicated to maintain a high level of proinflammatory cytokine production compared with M_2a_Фs and M_2c_Фs [32]. While CCL17, CCL22, and CCL24 were exceptions in the inflammatory gene profile, they were more abundant in M_2a_Ф exosomes. This result was also consistent with the cytological characteristics of M_2a_Фs [32, 33]. These factors are engaged in numerous pathophysiological processes, including the interface of immunity, tissue homeostasis, and metabolism, and are an important part of the functional specificity of MФ subtypes. The results indicated that exosomes contain certain genes from their source cells and maintain consistent expression in the cytoplasm. Although further studies are required to determine whether the transferred mRNAs are translated into proteins in recipient cells, the present results indicated that exosomes may be involved in the biological actions of MФs.

In the functional analysis, mRNAs detected in M_2b_Ф exosomes were predicted to affect biological pathways, including the TLR, TNF, NLR, and NF-*κ*B signaling pathways, in recipient cells. The TLR and NLR signaling pathways are widely involved in innate and adaptive immunity by binding pathogen-associated molecular pattern molecules (PAMPs) and damage-associated molecular pattern molecules (DAMPs) [34, 35]. The TNF signaling pathway governs immune system development, cell survival, and proliferation and regulates metabolic processes [36]. In addition, as a proinflammatory cytokine, TNF mainly induces apoptosis in target cells as well as proteolytic processes [37]. The activation of the TLR and TNF signaling pathways triggers the release of the transcription factor NF-*κ*B to the nucleus, finally influencing a broad range of biological processes [38]. Furthermore, the transferred mRNAs in M_2b_Ф exosomes probably participate in autoimmune and infectious diseases, including rheumatoid arthritis, pertussis, malaria, leishmaniasis, legionellosis, amoebiasis, salmonella infection, and Chagas disease. These results are consistent with the results of Mosser et al. on M_2b_Ф [17, 39]. He considers M_2b_Фs, or regulatory MФs, to be a category of MФs whose major physiological role is to dampen inflammatory immune responses and prevent immunopathology. In this regard, M_2b_Фs are certainly distinct from classically activated MФs and quite different from MФs treated with the type 2 T-helper cell (TH2) cytokines IL-4 or IL-13, the so-called M_2_Фs.

In summary, in the present study, the mRNA transcriptomes of exosomes from three subtypes of M_2_Фs were characterized. Bioinformatics analyses demonstrated that the mRNAs contained in the exosomes were mediators of the functions of their source cells. Furthermore, it was proved that M_2b_Фs are unique among M_2_Фs and are distinctly different from M_2a_Фs and M_2c_Фs. Regulatory MФs is probably a more appropriate term for this subtype. Further functional investigations based on these results may help to advance the understanding of the physiological roles of exosome-transferred mRNAs in MФ functions.

There are some limitations of this study. First, though macrophage populations were polarized according to the methods suggested in the guidelines [40], we could not know for sure the purity of each M_2_Ф population by using flow cytometry or immunofluorescent staining. And we did not have completely pure populations for all studies. Part of the reason is that certain cell-surface markers for each population are lacking [40]. An example of problematic marker use is the expression of CD163 as a “marker” for M_2c_Ф, which has led to interpretive problems because CD163 is also induced in M_2a_Ф [16]. Secondly, the functions of the abundant mRNAs in exosomes were concluded by KEGG pathway enrichment analysis based on the overlap of individual molecules and were not accurate and precise enough.

## Figures and Tables

**Table 1:** Primer sequences used for RT-PCR.

Gene (rat)	Forward	Reverse
GAPDH	GGTCATCCATGACAACTT	GGGGCCATCCACAGTCTT
CCL17	AGTGCTGCCTGGACTACTTC	CTGGACAGTCTCAAACACGATGG
CCL1	GAGCCTGCAGTTTCACTCA	GATCTGTGAGCCTGCATCAGT
CXCL13	ACATGCCTAGACTGAGAGCT	AAGGCAGATGGCCAGTAGAAG

CCL: C-C motif chemokine ligand; CXCL: C-X-C motif chemokine ligand.

**Table 2:** Unique mRNAs in exosomes of different group.

Symbol	Description
Unique mRNAs in Exo-M2a	
*Alpk2*	Alpha-kinase 2
*Cd55*	CD55 molecule, decay-accelerating factor for complement
*Cnksr1*	Connector enhancer of kinase suppressor of Ras 1
*Dmd*	Dystrophin
*Dok5*	Docking protein 5
*Edn1*	Endothelin 1
*Fam26d*	Family with sequence similarity 26, member D
*Hey1*	hes-related family bHLH transcription factor with YRPW motif 1
*LOC103690049*	Deoxyribonuclease gamma-like
*Maob*	Monoamine oxidase B
*Timd2*	T-cell immunoglobulin and mucin domain containing 2
*Xpnpep2*	X-prolyl aminopeptidase 2
Unique mRNAs in Exo-M2b	
*Cdo1*	Cysteine dioxygenase type 1
*Gcnt2*	Glucosaminyl (N-acetyl) transferase 2, I-branching enzyme
*Gpr84*	G protein-coupled receptor 84
*Il2ra*	Interleukin 2 receptor subunit alpha
*LOC102550416*	Small proline-rich protein 2I-like
*LOC103692265*	Killer cell lectin-like receptor 4
*LOC103692615*	Uncharacterized LOC103692615
*LOC689205*	Similar to cytoplasmic beta-actin
*Nrarp*	Notch-regulated ankyrin repeat protein
*Podnl1*	Podocan-like 1
*Ptgs2*	Prostaglandin-endoperoxide synthase 2
*Serpinb6b*	Serine (or cysteine) peptidase inhibitor, clade B, member 6b
*Snhg4*	Small nucleolar RNA host gene 4 (nonprotein coding)
Unique mRNA in Exo-M2c	
*Slc35g1*	Solute carrier family 35, member G1

**Table 3:** Top 30 different abundant mRNAs in exosomes between groups.

Exo-M2b vs. Exo-M2a		Exo-M2a vs. Exo-M2c		Exo-M2b vs. Exo-M2c	
Gene	log2(FC)	Gene	log2(FC)	Gene	log2(FC)
Upregulation					
*LOC689205*	9.67	*Ccl24*	8.48	*LOC102550257*	8.76
*Il12a*	7.81	*Hba2*	8.46	*Ccl20*	8.62
*LOC103690054*	7.25	*F7*	8.43	*F10*	8.46
*Cxcl2*	7.23	*Fbp1*	8.22	*Mmp3*	8
*Ppap2c*	7.22	*Maob*	7.97	*Cpxm1*	7.51
*Htra1*	7.15	*Prm2*	7.91	*Cxcl6*	7.29
*Gcnt2*	7.08	*Plet1*	7.3	*Ptges*	7.08
*Csf2*	6.95	*Ccl17*	7.1	*Mmp13*	7.07
*Ch25h*	6.89	*Wfs1*	6.98	*Mri1*	7.01
*LOC100359515*	6.85	*RGD1308147*	6.97	*Csf2*	6.96
*Scin*	6.81	*Il1r2*	6.9	*Tut1*	6.95
*LOC102550257*	6.75	*Mri1*	6.83	*Adora2a*	6.93
*Gpr84*	6.74	*Ptrf*	6.77	*Cited4*	6.84
*Cpxm1*	6.65	*Uchl3-ps1*	6.54	*Il12a*	6.82
Downregulation		*Ccl22*	6.13	*Scin*	6.82
*Maob*	-9.53	*Socs1*	5.57	*Gpr84*	6.76
*Fam57b*	-9.05	*Timd2*	5.26	*Il10*	6.73
*Gpr183*	-8.75	*Trmt6*	5.19	*Il1a*	6.69
*Timd2*	-7.91	*Slamf1*	5.17	*F3*	6.68
*Acsl3*	-7.83	*Ebna1bp2*	4.63	*Csf3*	6.6
*Ap1s3*	-7.67	*LOC102556076*	4.61	*Nupr1*	6.58
*Ccl22*	-7.67	Downregulation		*Cd69*	6.55
*Mylk3*	-7.52	*Pxmp2*	-7.57	*RGD1308147*	6.51
*Tnfaip8l3*	-7.51	*Decr2*	-6.68	*Cxcl1*	6.49
*Fbp1*	-7.45	*Tma7*	-6.42	Downregulation	
*Amica1*	-7.29	*Ccdc82*	-6.18	*Decr2*	-8.26
*Pet112l*	-7.14	*LOC100911372*	-5.44	*Samhd1*	-7.48
*Plet1*	-6.96	*LOC102552889*	-5.13	*Chn2*	-7.47
*LOC102556076*	-6.86	*Rab33b*	-4.85	*LOC102552889*	-7.16
*Npw*	-6.74	*LOC100910130*	-4.69	*Pet112l*	-6.50

log2(FC): log2(fold change); positive numbers: upregulation; negative numbers: downregulation.

## Data Availability

The data used to support the findings of this study are included within the article.

## Supplementary Materials

Supplementary Materials Figure S1 influence of different M_2_Ф subtypes, culture supernatants, and exosomes on cardiac fibroblasts (CFs). CFs were cocultured with MФs (A) or cultured with MФ supernatants (B) or exosomes isolated from MФ supernatants (C). The same volume of culture medium was used as a control (NT). The expression of collagen I (green) and *α*-smooth muscle actin (*α*-SMA) (red) in the CFs was measured by immunocytochemistry (200x). The expression of COL-1 and *α*-SMA was decreased by coculturing with M_2b_Фs and was increased by coculturing with M_2a_Фs or M_2c_Фs. The cell culture supernatants and exosomes showed the same effects as their source cells.
