# Supplementary material for: Characterization of mRNA Profiles of Exosomes from Diverse Forms of M2 Macrophages
Source: Biomed Res Int. 2020 Nov 21;2020:1585306. doi: 10.1155/2020/1585306 (PMC7704128; doi:10.1155/2020/1585306)
Supplement: Supplementary Materials — Figure S1 influence of different M2Ф subtypes, culture supernatants, and exosomes on cardiac fibroblasts (CFs). CFs were cocultured with MФs (A) or cultured with MФ supernatants (B) or exosomes isolated from MФ supernatants (C). The same volume of culture medium was used as a control (NT). The expression of collagen I (green) and α-smooth muscle actin (α-SMA) (red) in the CFs was measured by immunocytochemistry (200x). The expression of COL-1 and α-SMA was decreased by coculturing with M2bФs and was increased by coculturing with M2aФs or M2cФs. The cell culture supernatants and exosomes showed the same effects as their source cells. [file 1585306.f1.docx]

**Supplementary Data**


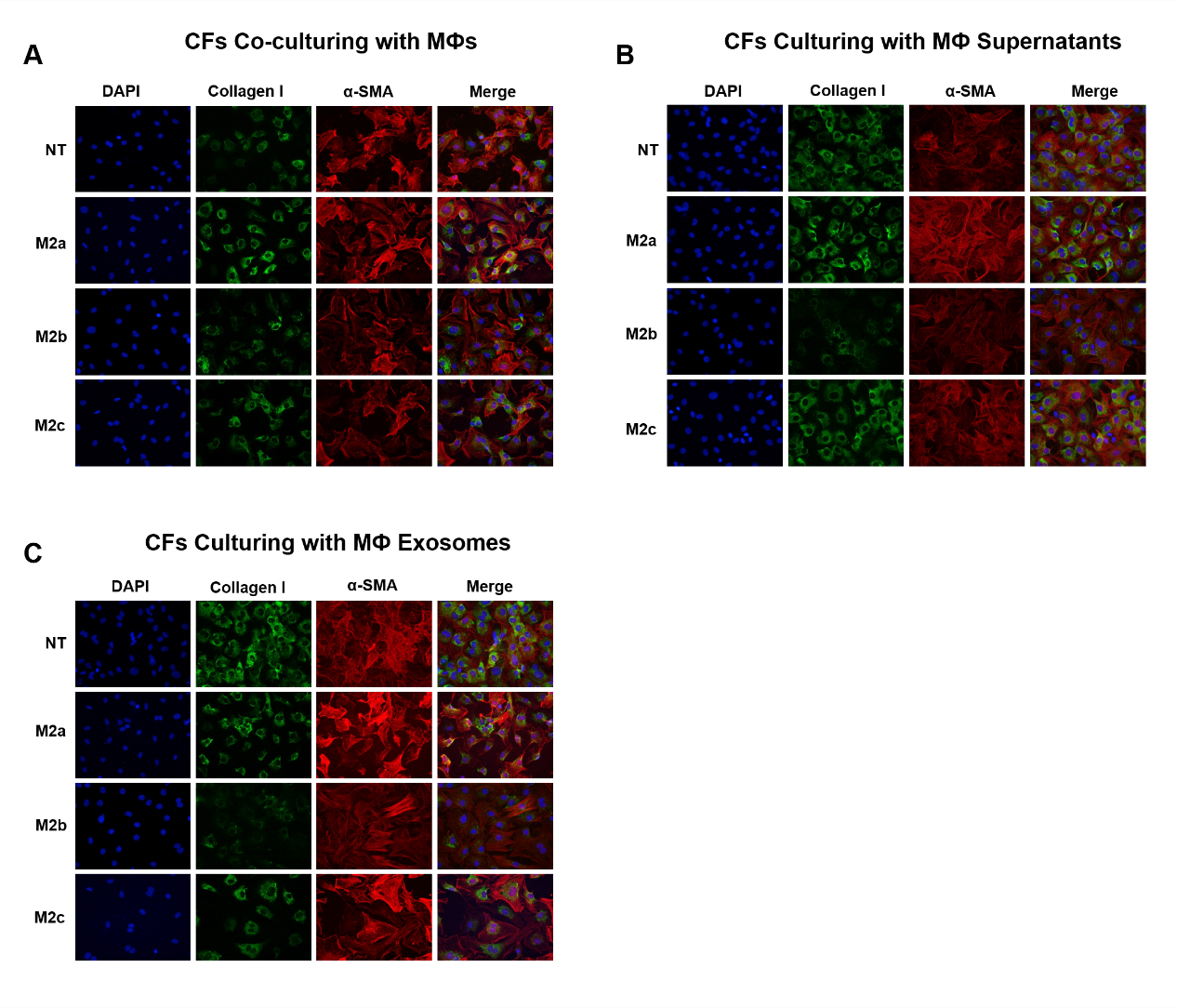


**Figure S1. Influence of different M_2_Ф subtypes, culture supernatants, and exosomes on cardiac fibroblasts (CFs).** CFs were cocultured with MФs **(A)** or cultured with MФ supernatants **(B)** or exosomes isolated from MФ supernatants **(C)**. The same volume of culture medium was used as a control (NT). The expression of collagen I (green) and α-smooth muscle actin (α-SMA) (red) in the CFs was measured by immunocytochemistry (200×). The expression of COL-1 and α-SMA was decreased by coculturing with M_2b_Фs, and was increased by coculturing with M_2a_Фs or M_2c_Фs. The cell culture supernatants and exosomes showed the same effects as their source cells.
